# Robust Arm Impedocardiography Signal Quality Enhancement Using Recursive Signal Averaging and Multi-Stage Wavelet Denoising Methods for Long-Term Cardiac Contractility Monitoring Armbands

**DOI:** 10.3390/s23135892

**Published:** 2023-06-25

**Authors:** Omar Escalona, Nicole Cullen, Idongesit Weli, Niamh McCallan, Kok Yew Ng, Dewar Finlay

**Affiliations:** School of Engineering, Ulster University, Belfast BT15 1AP, UK

**Keywords:** armband ICG sensing methods, impedance cardiography, Arm-ICG signal enhancement, recursive signal averaging, thorax impedocardiography, brachial-artery-based ICG surrogate, ambulatory hemodynamics, heart contractility monitoring, two-stage Daubechies wavelet denoising, arm stroke volume, signal-averaged ICG

## Abstract

Impedance cardiography (ICG) is a low-cost, non-invasive technique that enables the clinical assessment of haemodynamic parameters, such as cardiac output and stroke volume (SV). Conventional ICG recordings are taken from the patient’s thorax. However, access to ICG vital signs from the upper-arm brachial artery (as an associated surrogate) can enable user-convenient wearable armband sensor devices to provide an attractive option for gathering ICG trend-based indicators of general health, which offers particular advantages in ambulatory long-term monitoring settings. This study considered the upper arm ICG and control Thorax-ICG recordings data from 15 healthy subject cases. A prefiltering stage included a third-order Savitzky–Golay finite impulse response (FIR) filter, which was applied to the raw ICG signals. Then, a multi-stage wavelet-based denoising strategy on a beat-by-beat (BbyB) basis, which was supported by a recursive signal-averaging optimal thresholding adaptation algorithm for Arm-ICG signals, was investigated for robust signal quality enhancement. The performance of the BbyB ICG denoising was evaluated for each case using a 700 ms frame centred on the heartbeat ICG pulse. This frame was extracted from a 600-beat ensemble signal-averaged ICG and was used as the noiseless signal reference vector (gold standard frame). Furthermore, in each subject case, enhanced Arm-ICG and Thorax-ICG above a threshold of correlation of 0.95 with the noiseless vector enabled the analysis of beat inclusion rate (BIR%), yielding an average of 80.9% for Arm-ICG and 100% for Thorax-ICG, and BbyB values of the ICG waveform feature metrics A, B, C and VET accuracy and precision, yielding respective error rates (ER%) of 0.83%, 11.1%, 3.99% and 5.2% for Arm-IG, and 0.41%, 3.82%, 1.66% and 1.25% for Thorax-ICG, respectively. Hence, the functional relationship between ICG metrics within and between the arm and thorax recording modes could be characterised and the linear regression (Arm-ICG vs. Thorax-ICG) trends could be analysed. Overall, it was found in this study that recursive averaging, set with a 36 ICG beats buffer size, was the best Arm-ICG BbyB denoising process, with an average of less than 3.3% in the Arm-ICG time metrics error rate. It was also found that the arm SV versus thorax SV had a linear regression coefficient of determination (R^2^) of 0.84.

## 1. Introduction

According to the World Health Organisation (WHO) (2023) [1], cardiovascular diseases (CVDs) are the leading cause of death worldwide, amounting to approximately 17.9 million deaths in 2019, representing 32% of all global deaths, of which 85% were due to heart attacks and strokes. It is recognized that early diagnosis of such diseases can result in a better treatment plan, and subsequently, a better outcome [1]. Hypertension, myocardial ischemia and infarction, and many cardiac arrhythmias can be identified by abnormalities in a patient’s hemodynamic parameters [2]. Impedance cardiography (ICG) offers a non-invasive method to derive hemodynamic parameters from waveform analysis [3].

Traditionally, hemodynamic parameters, such as cardiac output (CO) and stroke volume (SV), are measured using methods such as pulmonary artery catheter (PAC) thermodilution (the gold standard). However, this method, as well as similar methods, are highly invasive and require highly trained staff. Other methods, such as magnetic resonance imaging (MRI) and Doppler echocardiography, are less invasive but are relatively expensive and require highly trained professionals to administer them [3]. ICG-based methods have eradicated the issues associated with obtaining patient hemodynamic parameters by introducing a non-invasive, relatively inexpensive and user-friendly alternative. Furthermore, ICG recordings enable continuous monitoring, giving a more accurate representation, compared with conventional methods, which are discontinuous [4].

**Figure 1:**
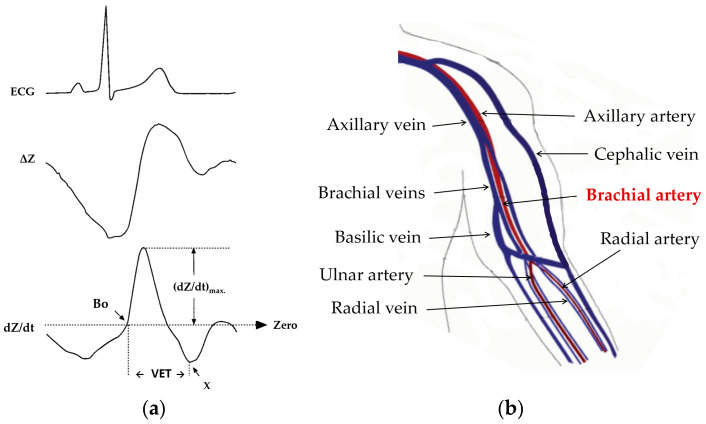
(**a**) Typical timing of ECG events, changes in impedance (ΔZ) and the rate of change of impedance with respect to time (dZ/dt); Bo is the zero-crossing prior to the ICG main peak. (**b**) Anatomical localisation of the brachial artery and other blood vessels along the arm.

Conventional ICG or impedocardiography sensing is achieved by means of the transthoracic bioimpedance approach, whereby electrodes are placed on a segment between the patient’s chest diaphragm and neck (Thorax-ICG), wherein a constant low-amplitude (<1 mA), high-frequency (30–100 kHz, typically) alternating current is applied [5]. Typical ICG recording waveform dynamics and relative timing with the electrocardiogram (ECG) signal is illustrated in Figure 1a. It is quite common to refer to the signal-conditioned dZ/dt derivative waveform as the ICG signal, as this processed waveform provides the key dynamic features for estimating CO and SV hemodynamic indicators. Furthermore, in recent years, the technique of acquiring an ICG signal from the arm (Arm-ICG) has received increasing attention [6,7,8,9], and more recently, in a study conducted in 2015, Saugel et al. [10] investigated cardiac output measured from the radial artery along the forearm. However, several issues related to noise interference and Arm-ICG signal quality associated with their approach have consistently been identified. In this study, Arm-ICGs were recorded along the left arm brachial artery.

The brachial artery is located in the upper arm and is an extension of the axillary artery (Figure 1b). It stretches along the ventral surface of the arm and then branches off at the forearm to form arteries such as the radial and ulnar arteries. It primarily functions to supply blood to the biceps, triceps and coracobrachialis muscles [11].

Stroke volume (SV) is defined as the volume of blood that is pumped from the left ventricle during a single systolic cardiac contraction [12]. According to Kubicek’s method for estimating SV [13], the ICG signal (or the dZ/dt waveform) provides the required signal amplitude and time metrics, namely, (dZ/dt)_max_ and the ventricular ejection time (VET), as depicted in Figure 1a. The product of these yields the maximum impedance change metric (ΔZ), which is linearly related to the heart (in conventional Thorax-ICG) or the brachial artery (in the Arm-ICG) blood volume change (ΔV) for every heartbeat (ΔV_THORX_ and ΔV_ARM_, respectively), where the constant of proportionality depending on the baseline characteristics (body dimension of electrically participating tissue parts and baseline impedances) specific to each subject [5,6,7]. Nevertheless, when deciding on the location of arm electrodes, the correct location along the brachial artery is important to consider for the strongest signal. The upper arm offers some advantages over the thoracic measurements as it is far away from the lung bioimpedance signal noise component [8].

From the estimated value of SV (cm^3^), the CO (L/min) can then be deduced from the product of SV and the heart rate (HR) of the patient [8]. These clinical indicators can be used to diagnose heart failure using various methods [14,15]. Simultaneous recording of the ICG and the electrocardiogram (ECG) signals can be used for advanced non-invasive diagnostic purposes [16].

When analysing the ICG signal, it is important to note that this technique can produce signals embedded in considerable noise, which is caused by factors such as motion artifact noises and an unstable electrode–skin interface. This can make the identification of certain ICG waveform features relatively difficult [7,14,17,18]. To address this problem, certain initial pre-filtering techniques are applied in order to achieve a basic level of signal quality enhancement. The linear digital Butterworth infinite impulse response (IIR) filter and the Savitzky–Golay (SG) filter are the most reported digital filters for the ICG signals prefiltering stage [7,14,19]. The SG digital filter has a finite impulse response (FIR) [20,21]. It was reported that the SG filter presents the lowest error rate impact on ICG waveform feature metrics extraction, which is relevant for determining the SV on a beat-by-beat basis [14]. In this comparative study, for both the conventional Thorax-ICG and novel Arm-ICG signals, prefiltering with a cascaded combination of both Butterworth and SG processes was adopted.

Further robust denoising methods are required for addressing Arm-ICG signal quality enhancement issues in such a way that makes the signal quality clinically acceptable and attractive for long-term ambulatory cardiac contractility (SV) monitoring for the benefit of cardiac patients. Based on previous knowledge of effective Arm-ICG and Arm-ECG denoising [7,22,23,24,25,26,27,28,29] methods, a satisfactory and robust solution was investigated by means of advanced processes, such as discrete wavelet transform (DWT)-based filtering techniques, supported by ensemble averaging algorithms for cardiac deterministic ICG events [30], both in the Arm-ICG and Thorax-ICG on a beat-by-beat (BbyB) basis. The widely recommended mother wavelet configuration used in DWT-based denoising approaches for ICG signals is the Daubechies wavelet, and more specifically, the fourth-order (Db4) and the eighth-order (Db8) Dabechies wavelets were reported to be suitable for the ICG signal [14,17,22,28,29]. Moreover, Chabchoub et al. [17] conducted an experiment to compare different wavelet families, i.e., Haar, Daubechie (Db), Symlet (Sym) and Coiflet. The results revealed that the Db wavelet with order 8 (db8) exhibited superior performance, surpassing the other wavelet families. Nonetheless, there is ongoing interest by some research teams who have reported the promising performance of the Sym wavelet for denoising the conventional ICG signals (Thorax-ICG) [31,32], and other studies reported the suitability of both the Db and the Sym wavelet families for other biomedical signals, such as EEG, EMG and ECG [33,34,35,36]. However, the refined question of which of these wavelets would be more suitable for denoising the Arm-ICG waveform characteristics on a BbyB basis needs to be addressed. Therefore, at this pilot research level, an initial step for addressing this refined question was taken by focussing on the Db wavelet family and investigating their optimisation for Arm-ICG signals denoising for the BbyB mode of operation due to its wide use in ECG and ICG signals filtering reported in the literature and the team’s expertise on using this particular wavelet family [22,28,29].

In this article, the materials and methods for simultaneous ICG and ECG recording modes and adequate ICG signal linear prefiltering design, including the SG filter, are described first. Then, the advanced data-driven denoising methods of optimized Db wavelet orders for arm and thorax ICG recording modes, assisted by the ensemble and recursive signal averaging processes to enable effective BbyB operation and the respective denoising performance and output ICG signal quality assessment metrics, are presented. This is followed by definitions of the four main ICG waveform features considered for this study, as well as the metrics for a functional relationship between arm and thorax ICGs assessment, associated with the effectiveness of Arm-ICG signal quality enhancement. In the Section 3, the results of the denoising performance and accuracy/precision of the ICG waveform features and SV measurements are presented. A discussion of the results is presented in Section 4, and the concluding remarks and acknowledgements are given in the final sections.

## 2. Materials and Methods

### 2.1. Data Acquisition and Sensor Systems

**Figure 2:**
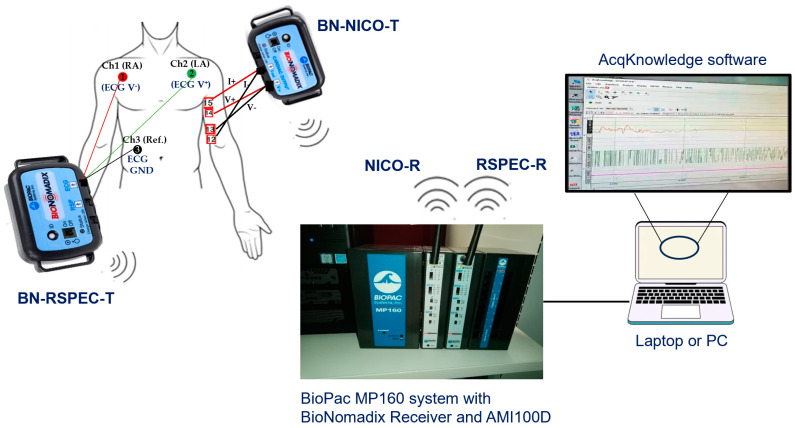
Simultaneous ICG and ECG acquisition channels hardware system block diagram.

Simultaneous recording of ICG and ECG signals was implemented using the BioPac MP160 Data Acquisition Hardware Systems and associated AcqKnowledge 5.0 software (BIOPAC Systems, Inc., Goleta, CA, USA) with two wireless front-end devices: BN-NICO-T for ICG signals and BN-RSPEC-T for ECG signal, as illustrated in Figure 2. These devices are digital RF transmitters (2.4 GHz) linked to NICO-R and RSPEC-R ECGs, respectively, while these receivers attached to the MP-160 central module are also connected to the laptop computer through an ethernet cable. It operates with a fixed bioimpedance constant AC source at 100 kHz frequency and 1 mA (rms) regulated amplitude.

### 2.2. ICG and ECG Recording Modes and Analysis Tools

Recorded ICG and ECG data from healthy subjects were taken while sitting and at rest for two modes of recording: (a) conventional Thorax-ICG and (b) experimental Arm-ICG. In each mode session, data recording was for 8 min (480 s) continuously, having their left arm resting on a desk whilst remaining silent and motionless. The recordings were taken at a sampling rate of 2000 Hz. Data signal processing was carried out using the Mathworks Matlab (9.9 R2021b) programming environment, and descriptive statistical analysis was carried out using MS Excel (within Microsoft 365).

### 2.3. Arm ICG Electrode Location

**Figure 3:**
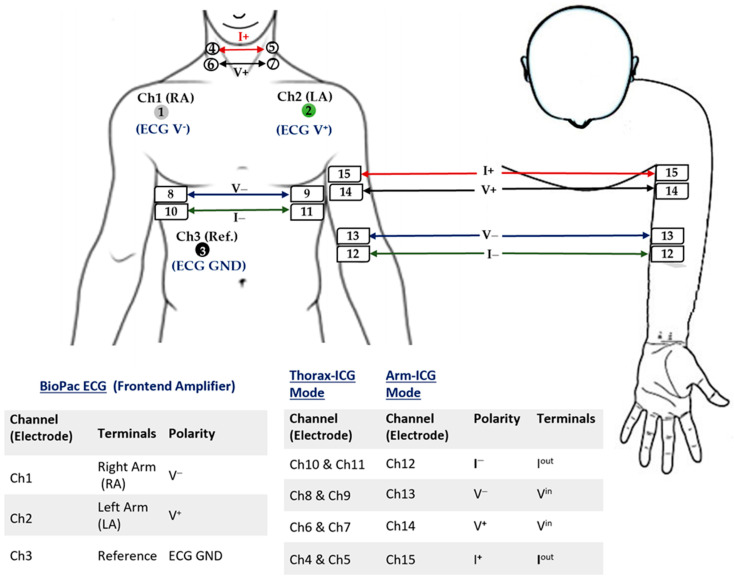
Electrode placement for the ECG standard Lead I, ICG-Thorax and ICG-Arm (upper arm).

The Arm-ICG electrode locations for both the bioimpedance current source and voltage sensing followed the expertise gained in previous work for sensing the strongest Arm-ICG signal along the ventral-inner surface of the upper left arm [7]. This approach enabled stable ICG signal quality pick up from the brachial artery systolic and diastolic blood volume changes, which are in proportional relation to the sensed arm impedance changes (ΔZ_ARM_), from which the Arm-ICG waveform is derived (see Figure 1a). Thus, current and voltage electrodes were placed along the same arm axial line from near the axilla point to the lower end of the upper left arm, as depicted in Figure 3, and the electrodes had a longitudinal placement to mimic the parallel electrode arrangement for the conventional Thorax-ICG (also shown in Figure 3).

### 2.4. ICG Pilot Study Data Set

Simultaneous ICG and ECG recordings were performed on different days on 15 healthy volunteer subjects aged between 22–65 years, with a proportion of 53.3% male. Each subject case and mode recording (conventional Thorax-ICG and experimental left Arm-ICG modes) were 8 min long (480 s) at a data sample rate of 2000 Hz. Conventional electrodes (MBS-3BF3 Disposable Ag/AgCl ECG/EMG Monitoring Electrodes, Multi Bio Sensors Inc., El Paso, TX, USA) gelled with highly conductive, hypoallergenic electrode gel (Signa gel, Parker Laboratories, Inc., Fairfield, NJ, USA) were used for both the ICG and ECG recordings (two simultaneous channels for each ICG recording mode). The electrodes were placed as illustrated in Figure 3. Channels 1 to 3 were for recording the ECG (chest standard Lead I; in any ICG recording mode), channels 4 to 11 were for recording the Thorax-ICG, while channels 12 to 15 were for recording the left upper Arm-ICG (axially along the arm). The recorded data from the 15 volunteers were managed using the BioPac AcqKnowledge 5.0 software and exported as Matlab data files (.mat). Table 1 summarises the main baseline demographic characteristics for the subjects (N = 15) recorded and included in this pilot study, which were fully compliant with the project’s set protocol.

The in-house (Ulster University) study protocol ethics and research governance review was considered and approved by the local Health Research Ethics Filter Committee panel on 6 January 2023 (project application reference number: FCNUR-22-092-A).

Table 1 presents a summary of the baseline demographic characteristics of the 15 included research subjects in this pilot investigation. There the characteristics of a good representative sample of the healthy population can be observed, with a median age of 39 and an interquartile range of 23 years. Furthermore, there was a reasonably balanced gender representation: 8 males (53.3%) and 7 females (46.7%). The body dimensions are within a relatively narrow margin: average height of 167 cm ± 8.5 cm (SD), chest of 93 cm ± 8 cm (SD), and average BMI of 25.2 ± 3.9 (SD) kg/m^2^. Important characteristics for this study, as they enable the calculation of the stroke volume (SV) according to Kubicek’s SV formula [13], are the thorax and arm L distances of the thorax and arm participating tissue and the baseline off-set impedance Zo, which are specific to each subject.

### 2.5. ICG and ECG Prefiltering Stage with IIR Filters

The recorded raw ICG and ECG signals output from the above-described BioPac system were presented at a wide bandwidth of 100 Hz and for this ICG monitoring study, and both raw ICG and ECG acquired signals were subjected to low pass filtering with an at least 40 Hz cut-off frequency. The ICG signals in both recording modes (Thorax-ICG and Arm-ICG) were conditioned using a pre-filtering process to significantly reduce the noise artefacts without deteriorating the main spectral content of the ICG signals, which were expected to be not wider than the T-wave in the ECG for monitoring purposes. Therefore, as in a previous study [7], the ICG signals pre-filtering stage consisted of 8th-order low-pass Butterworth filtering at an 8 Hz cut-off frequency, followed by high-pass, 4th-order filtering at a 0.5 Hz cut-off frequency. The ECG signals provided by the BioPac ECG module were simply low-pass-filtered at 40 Hz with a second-order Butterworth filter.

### 2.6. Savitzky–Golay Prefiltering of the ICG Signals Stage

The Savitzky–Golay (SG) smoothing filter is considered a type of finite impulse response (FIR) digital filter [17,19], which is represented by polynomial equations. Based on the least squares method [20], the SG filter is typically used to smooth a noisy signal whose frequency range of the signal without noise is large. In this type of application, the SG filter performs better than the standard FIR filters because these tend to attenuate a significant portion of high frequencies of the signal and the noise. Although the SG filter is more effective in preserving relevant high-frequency signal components, SG filters are less effective in removing high-level noises in a signal [37]. The particular formulation of the SG filter preserves moments of higher orders much better than other methods. As a consequence, the widths and amplitudes of the peaks for the desired signals tend to be preserved [14,20]. The SG filter can be considered to have input data that are denoted as an N-dimensional vector ***x***:

(1) $$x={\left[x_{-M},\ldots ,x_{-1},x_{0},x_{1},\ldots ,x_{M}\right]}^{T}$$

where *M* is a natural number that denotes points evenly distributed on both sides of a central point $x_{0}$ and *N* = 2*M* + 1. A polynomial of degree *d* is then fitted using the *N* data samples of ***x*** using

(2) $${\hat{x}}_{m}=c_{0}+c_{1}m+\cdots +c_{d}m^{d}\;,\;\;-M\;\leq \;m\;\leq \;M$$

where ${\hat{x}}_{m}$ represents the *m*th sample of the smoothed data. Next, *d* + 1 polynomial basis vectors $s_{i}$ are defined using

(3) $$s_{i}\left(m\right)=m_{i},\;\;-M\;\leq \;m\;\leq \;M$$

where the matrix *S* has $s_{i}$ columns and a size of N×(d+1), with the columns represented by $S=\left[s_{0},\;s_{1},\ldots ,s_{d}\right]$. The smoothed values can be expressed as a vector $\hat{x}=Bx$ using

(4) $$\hat{x}=Bx=\overset{d}{\underset{i=0}{\sum }}c_{i}s_{i}$$

where *B* is a matrix created by combining the polynomial basis vectors $s_{i}$ with the coefficients $c_{i}$. The value $y_{0}$, which represents the first smoothed data sample ${\hat{x}}_{0}$, is determined based on the centre of the filter $b_{0}:\;y_{0}=b_{0}^{T}x$ using

(5) $$y_{0}=b_{0}^{T}x=\sum_{m=-M}^{M}\;b_{0}\left(m\right)x_{m}$$

where the filter coefficients at the centre are denoted by $b_{0}$. The *N*-dimensional vector ***x*** can be shifted by *n* instants of time, as denoted by

(6) $$x\;\to {\left[x_{n-M},\ldots ,x_{n-1},x_{n},x_{n+1},\ldots ,x_{n+M}\right]}^{T}$$

The SG filter produces the output for smoothing noisy data using

(7) $$y\left(n\right)=\sum_{m=-M}^{M}b_{0}\left(-m\right)\;x\left(n-m\right)$$

where $b_{0}$ represents the filter coefficients. By performing *I* differentiations on Equation (7), the generic form of the filter output is derived using

(8) $$y^{i}\left(n\right)=i!\sum_{m=-M}^{M}g_{i}\left(-m\right)\;x\left(n-m\right)\;\;\;i=0,1,\ldots ,d$$

where $g_{I}$ denote the coefficients of the differentiated filter, which may not necessarily exhibit symmetry. The use of derivatives is a usual manipulation when the removal of signal offsets is needed during the pre-processing phase [19]. Usually, the 1st derivative (*I* = 1) removes the systematic offsets of the signal, while the 2nd derivative (*I* = 2) can eliminate linear variations.

For selecting the SG filter order in this study, knowledge of the intrinsic and relative ICG main pulse waveform characteristics previously reported [7] were considered. Therefore, the SG order was fixed for both modes of ICGs, namely, the thorax and arm, but because the Arm-ICG waveform has narrower pulse width characteristics than the Thorax-ICG, the SG filter order suitability was checked with Arm-ICGs. In doing so, tests of the comparative denoising performance for different polynomial orders were undertaken in some Arm-ICG signal cases. The trial polynomial orders ranged from 2 to 8. The exploratory results indicated that the SG filter order 3 provided the best denoising performance, which was in accordance with the recommended SG order for ICG denoising by other research groups [14].

Currently, there is a knowledge paucity of a suitable setting procedure for the SG filter size (N samples) for effective denoising ICG signals. Therefore, criteria were based on the time span of the main ICG pulse waveform feature being targetted for quality enhancement; the VET time width value (see Figure 1a) was investigated for an adequate size of the SG filter for this purpose, and a simple equation for determining a suitable value of N based on a priori average VET characteristic of the Thorax-ICG (relatively low noise, after simple IIR prefiltering as described in Section 2.5) was proposed. For this, the average measured VET value of 10 cases in this study was estimated as 248.3 ms ± 16.1 ms (SD) (see Table 2), which, at a 2000 Hz data sampling rate, yielded VET_MEAN_ ≈ 497 samples. However, an effective optimal SG filter size (N_OPT_) for ICG denoising was empirically determined by scaling the VET_MEAN_ (in samples) by a factor of ($1/\sqrt{2}$), or 0.71. Hence, the following proposed empirically deduced equation for an effective GS filter size was

(9) $$N_{\mathrm{OPT}}\;\approx \;{\mathrm{VET}}_{\mathrm{MEAN}}/\sqrt{2}$$

Therefore, the proposed empirical Equation (9) was used for the SG filter’s suitable size setting in this study: N ≈ 351 samples.

### 2.7. Beat-by-Beat Recursive Ensemble Averaging Denoising Process

#### 2.7.1. Beat-by-Beat Segmentation Algorithm

Beat-by-beat (BbyB) segmentation of each subject case for the simultaneous ICG and ECG recordings (for both arm and thorax ICG modes) was based on the single fiducial point (SFP) technique previously reported [7,23,24], which was applied to the ECG signal recorded in a particular ICG mode and subject case as the reference signal for extracting the accurate time position of valid ventricular depolarisation events of QRS complexes along the whole ECG recording. Therefore, the output from the SFP process is a vector of sample numbers (*n*) of valid heartbeat QRS complexes at the SFP position along the 480 s simultaneous ICG and ECG recording length. Thus, the heartbeat sample positions SFP vector was used for the accurate segmentation of both the simultaneous ICG and the ECG signals by defining 1400 samples (700 ms) centred around each sample point (element) in the SFP vector [23].

#### 2.7.2. ICG Ensemble Averaging Process

Ensemble averaging or signal-averaged ICG (SAICG) of segmented 700 ms ICG frames generated by every heartbeat sample position in the SFP vector was implemented over all detected and validated heartbeats along the 480 s ICG recording (arm or thorax ICG), the number of which (Nb) ranged between 400 and 700 beats, depending on the heart rate and quality of the respective chest ECG Lead I recording used for the SFP process [7]. This process provided the SAICG absolute reference of the best denoised ICG frame, or “noiseless” ICG signal against which any denoising process performance can be assessed on a BbyB basis [24].

#### 2.7.3. ICG Recursive Averaging BbyB Process

This BbyB denoising process presents updated denoised 700 ms ICG frames for every incoming heartbeat (Rq) processed by the SFP algorithm, which may be implemented analogically and in real time [25,38], recursively included in an ensemble averaging buffer order (Nb) of previous adjacent heartbeat ICG frames and updated in a first-in-first-out (FIFO) order.

The recursive averaging (Rav) process may be described iteratively as follows:

Define *K* as the total number of valid heartbeat ICG events in the whole ICG(*n*) recording data array (*n* = 1, 2, 3, …, 960,000 samples for the 480 s recording), which were simultaneously recorded with the standard chest ECG Lead I for a particular subject case and mode (Arm-ECG or Thorax-ICG), and SFP(*k*) is the associated SFP vector of the sample position (*n*) of consecutive valid heartbeat ICG events Rq(*k*), with *k* = 1, 2, 3, …, *K*, and *Nb* the order of the Rav process, which is the number of previous ICG beats 700 ms frame vectors included in the ensemble averaging buffer; e.g., we explored Rav denoising processes performance for *Nb* values of 16, 36 and 64. Then, for the current incoming heartbeat Rq(*k*), the 1400 samples (700 ms) output frame vector from the Rav(*k*) BbyB denoising process may be expressed as follows:

(10) $$\mathrm{Rav}(k)\;=\;\frac{\sum_{k=k-Nb}^{k=k}\;\mathrm{ICG}\left(\left(\mathrm{SFP}\left(k\right)-600\right):\left(SFP\left(k\right)+799\right)\right)}{Nb}$$

From this expression for the Rav denoising process, it is evident that the signal-averaged ICG (SAICG) over all detected and valid heartbeats along the 480 s ICG recording for obtaining the “noiseless” reference ICG 700 ms frame vector corresponds to the particular Rav(*k*) process having *Nb* set to (*K* − 1) beats.

#### 2.7.4. Selection of the Number of Beats Order (Nb) of the Rav Process

As the Rav denoising process is intended to be a practical BbyB, data-driven [25] and clinically valuable cardiac contractility monitoring tool, setting a convenient order value for *Nb* would be a trade-off decision between real-time tracking of fast changes in cardiac conditions, e.g., monitoring SV variations, and the acceptable signal quality enhancement level needed to enable reliable ICG metric measurements, particularly for Arm-ICG monitoring methods. Therefore, from Equation (4), the effects of large *Nb* values on the Rav process are both slowness and ICG quality enhancement, limited to a maximum of *Nb* = (*K* − 1).

With ICG recordings of 480 s in this study, of particular interest are *Nb* values associated with a relatively small-time proportion of the recording time, say around 10% of it, or less. Therefore, to investigate the relative signal quality enhancement of the Rav process for 3 convenient values of *Nb*, namely, 16, 32 and 64, the process was applied to simultaneous ECG signals from the chest (standard Lead I) and arm (bipolar Lead-1) database that was readily available from a previous investigation [22]. The outcomes of this exploratory investigation with 9 cases (sample size) are presented in Table 3. There, the relative ECG signal quality enhancement, arm versus chest, is reflected by the mean SNR_dB_ figure difference between the chest and arm using the performance metric SNR_diff_ (dB). The *Nb* value that achieved a lower SNR_dB_ difference between the chest control SNR_dB_ and the arm-SNR_dB_ indicates better comparative signal quality enhancement with respect to the control chest ECG signal quality. Based on these figures and the above cardiac changes fast-tracking trade-offs, the selected value of *Nb* was set to an intermediate value of 36 for this Arm-ICG study.

### 2.8. Beat-by-Beat Daubechies Wavelet Transform-Based Denoising of ICG Signals

**Figure 4:**
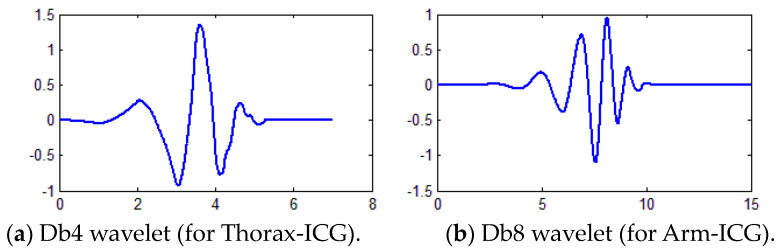
Illustration of the form pattern of the Daubechies wavelets adopted for (**a**) Thorax-ICG (Db4) and (**b**) Arm-ICG (Db) 2-stage wavelet denoising processes in this study.

An alternative, widely adopted, data-driven and advanced denoising process based on DWT filtering methods was investigated and a suitable approach for enabling DWT denoising on a BbyB basis was proposed. The proposed wavelet denoising process is a refinement of the previously reported 2-stage 4th-order Daubachies (Db4) mother wavelet method [22,29], which is applied for denoising simultaneous chest-ECG and Arm-ECG recordings. In a similar approach for this ICG denoising study, the Db4 wavelet was chosen since it is considered the best wavelet for the treatment of ICG signals, and its shape (Figure 4a) is similar to the observed shape of Thorax-ICG signal SAICG 700 ms frames [14,22]. However, it was observed in a previous study that the Arm-ICG SAICG 700 ms frames reveal a more convoluted, narrower main ICG pulse waveform (C metric) [22] and have a slightly higher frequency content than the Thorax-ICG. Hence, the Arm-ICG waveform tends to present more features that resemble the 8th-order Db8 wavelet (Figure 4b), and thus, an exploratory investigation of Db4 versus Db8 wavelet BbyB denoising of Arm-ICGs showed a preference for adopting the Db8 wavelet for the Arm-ICG wavelet denoising (WavDb8) process, as the Db8 was also recommended for ICG wavelet denoising by other research groups [17].

**Figure 5:**
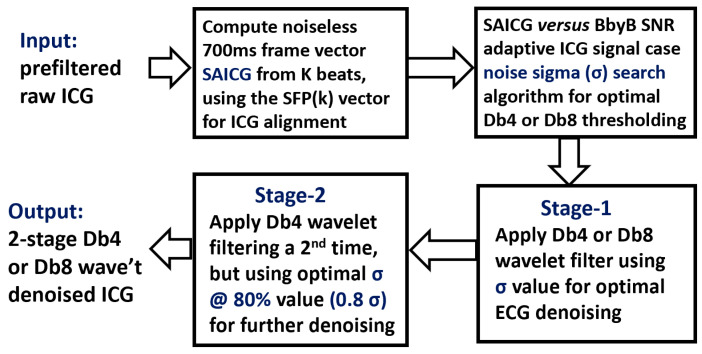
Algorithm block flowchart for the 2-stage ICG Db4/Db8 denoising process.

Furthermore, for enabling the Db4 and Db8 2-stage processes to operate on a BbyB basis in this study, after the ICG signal segmentation, a Tukey tapered cosine operation was applied to each heartbeat ICG 700 ms frame as described above. Then, their required thresholding task was supported by ensemble averaging algorithms as previously reported for arm-ECG wavelet denoising [22]; refined for this ICG study in an adaptive way [30] for each ICG subject and recording mode, namely, Arm-ICG and Thorax-ICG; and executed on a BbyB basis, as depicted in the Figure 5 block diagram.

The ICG beat 700 ms (1400 points) frame vector data was up-sampled (interp) by 2 (2800 points) before applying a wavelet filtering process and then the output data were down-sampled (decimated) back by 2 (back to 1400 points filtered vector). This manipulation enabled adequate use of the following widely used thresholding criterion:

*Thr* = *x* × σ (11)

where σ is the raw input ICG noise standard deviation level and

(12) $$X=\sqrt{2\times Log\left(n\right)}$$

where *n* is the number of data points of the ICG(*n*) input signal vector to the wavelet denoised process after up-sampling (interpolating) by a factor of two [22].

**Figure 6:**
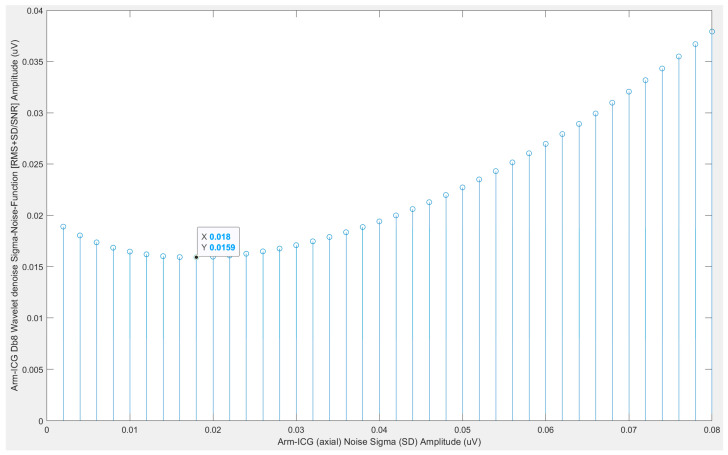
Optimal value of sigma (σ) for finding the algorithm output, i.e., sigma-NF(σ), for the Db8 wavelet denoising process of Arm-ICG, using subject case 4 as an example. The optimal σ (*x*-axis) value (0.018 mV) corresponded to the minimum value of the sigma-NF(σ) amplitude (*y*-axis) of 0.0159 mV in this case and mode (Arm-ICG).

The ICG signal noise component is defined as the 700 ms frame vector arrays difference of the SAICG (signal) and the incoming ICG beat Rq(*k*) for all beats *k* = 1, 2, …, *K*. Hence, the noise (*k*) vector is generated, and this is used by the noise sigma (σ) search algorithm to generate the noise function, NF(σ), calculated as: (noise__RMS_ + noise__SD_)/SNR, where SNR = (SAICG__RMS_)/(noise__RMS_) for a range of thresholding sigma values input into the wavelet denoising process to adaptively determine the optimal thresholding sigma value that minimises NF(σ) [22], as illustrated in Figure 6.

As a preliminary task for this study and to illustrate the output from the above-described wavelet thresholding optimisation method for estimated ICG signal noise standard deviation (sigma, σ) values, the wavelet optimal sigma (σ_optim_) for determining the thresholding value was found using Equations (11) and (12) for each mode of ICG signals, namely, the Thorax-ICG (Db4) and Arm-ICG (Db8) denoising processes, and for each of the 15 subject cases were determined as the output using the 2nd block process in Figure 5, and are presented in Table 4.

**Figure 7:**
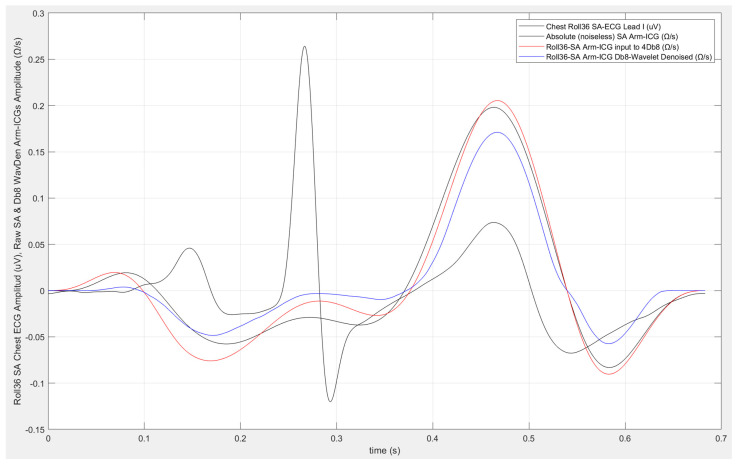
Example subject case 4 at beat #639 in a BbyB denoising process of the Arm-ICG: simultaneous ECG (mV) trace (black), noiseless Arm-ICG (Ω/s) 700 ms reference vector (black), plain Rav36 denoising process output ICG (Ω/s) 700 ms vector (red), and Db8-wavelet and Rav36 denoising process output ICG (Ω/s) 700 ms vector (blue).

Figure 7 illustrates the processed 700 ms frame vector output at beat number 639 for the example case 4 considered in the Figure 6 wavelet Db8 σ_optim_ determination plot, with the values indicated in Table 4 in the Arm-ICG recording mode, including the 36-beat Rav (Rav36) BbyB denoising process output frame vector for Rq(639) (red trace) and output for the cascaded combination of both denoising processes (blue trace), namely, Rav36 followed by the 2-stage Bb8 wavelet (WavDb8) process, for their qualitative comparison of denoising effects on the Arm-ICG waveform at that particular beat number *k* = 639. The SAICG reference “noiseless” vector is the ICG waveform black trace. The SAECG vector (also as a black trace) is included for the cardiac heartbeat event timing reference.

### 2.9. ICG Beat-by-Beat Denoised ICG Waveform Quality Assessment and Inclusion Criteria

#### 2.9.1. Pearson Correlation (*p*) BbyB with “Noiseless” SAICG Vector Reference

In this BbyB process, the maximally denoised SAICG 700 ms frame vector reference was considered as the control template for the targeted ICG signal quality against every incoming valid heartbeat ICG 700 ms frame vector, namely, Rq(*k*), in a BbyB basis waveform correlation assessment for a particular denoising process. Thus, the Pearson correlation coefficient (*p*) was used as a metric of similarity with the ICG signal SAICG control template. The conventional equation for the Pearson correlation coefficient percentage metric *p*(%) used in this study was the following one:

(13) $$p\left(\%\right)=\frac{\sum_{i=1}^{n}\left(Xi-\bar{X}\right)\left(Yi-\bar{Y}\right)}{\sqrt{\sum_{i=1}^{n}{\left(Xi-\bar{X}\right)}^{2}}\;\sqrt{\sum_{i=1}^{n}{\left(Yi-\bar{Y}\right)}^{2}}}\times 100$$

where $\bar{X}$ and $\bar{Y}$ and are the mean values of the *Xi* and *Yi* variable samples [22].

**Figure 8:**
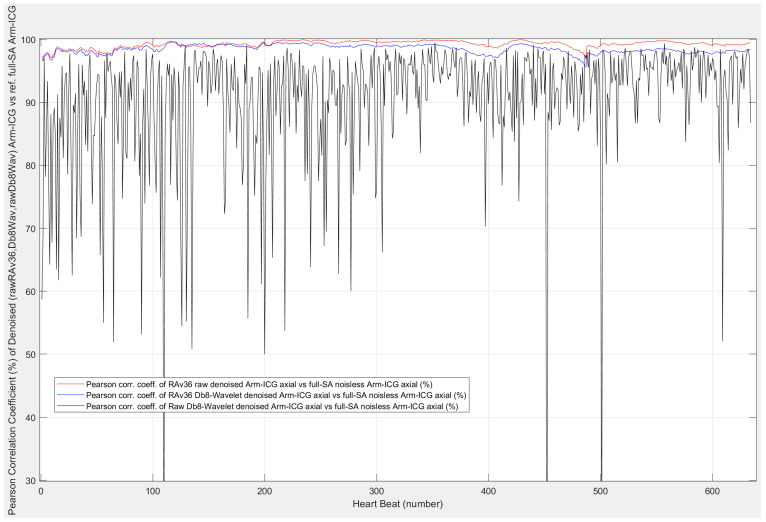
Pearson correlation coefficient *p*(%) of the denoised Arm-ICG (case 4) on a BbyB basis versus the noiseless Arm-SAICG vector.

Figure 8 illustrates a plot of the above-described Pearson correlation assessment of the denoised ICG beats on a BbyB basis, i.e., correlation *p*%(*k*) values for every heartbeat number (*k*) along the 480 s Arm-ICG recording for the same example case 4 considered in Figure 6 and Figure 7. For this case, the denoising performance on a BbyB basis of three ICG filtering processes can be appreciated: for the WavDb8 wavelet filtering (black), the 36 beats Rav36 recursive averaging (red), and the Rav36 + WavDb8 cascaded combination of the two (blue). Furthermore, the mean value of the generated *p*%(*k*) vector provides a simple and effective denoising performance metric for each filtering process under investigation. Another effective performance metric used for this study was found by setting *p* above 95% as the beat inclusion criteria and counting all the beats included (IncBt) out of the total number of valid beats (K) in the 480 s ICG recording, and then determine the beat inclusion rate percentage (BIR%) performance metric using BIR% = (IncBt/K) × 100.

This ICG waveform quality assessment method is quite versatile and can be used to detect bad-quality Arm-ICG episodes of ICG beats since they present *p*(%) values below a certain threshold value (e.g., *p* < 95%), and thus, can be classified as noisy episodes, which could be excluded for ICG features metrics determination, and hence, reducing their measurement error rate in ambulatory scenarios.

#### 2.9.2. BbyB Residual Noise and SNR Metrics Based on the SAICG Signal-Vector

Having defined the SAICG 700 ms frame vector as the ICG signal denoising target reference, any denoising process output vector difference from the SAICG reference ICG noiseless signal vector can be considered as the residual noise vector (noise), with an amplitude characterised by its RMS value (mV) or/and by its SD value (mV), which can be estimated on a BbyB basis to generate the noise_rms_(*k*) and the noise_sd_(*k*) vectors. Thus, the following definitions of noise and signal were used for this study:

**noise** = [SAECG “noiseless” 700 ms_vector frame] − [filter process output vector frame] and the absolute, noiseless reference signal 700 ms vector is defined as the signal, (14)

**signal** = [SAECG 700 ms frame obtained by ensembled averaging all valid ICG beats] (15)

A similar noise estimation procedure can be applied to the raw ICG input vector to the denoising process, and noise reduction figures, expressed in decibels, can be used as a denoising performance metric. However, for this study, the following definition of the signal-to-noise ratio (SNR) was adopted as a BbyB denoising performance metric [14,17]:

(16) $$\mathrm{SNR}=\frac{mean\;\left({\mathrm{signal}}^{2}\right)}{mean\left({\mathrm{noise}}^{2}\right)}$$

### 2.10. ICG Waveform Basic Features Metrics Definitions

**Figure 9:**
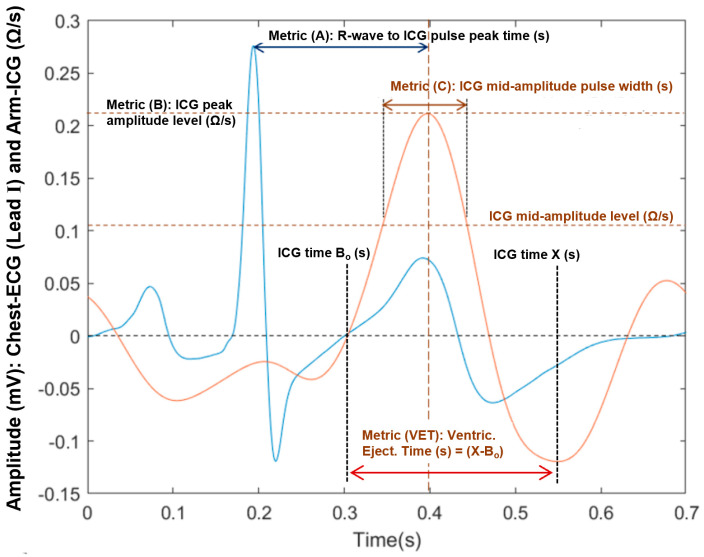
ICG waveform features A, B, C and VET metrics defined in the 700 ms ICG beat frame.

To further assess the ICG quality enhancement performance, the accuracy (error rate) and precision (e.g., SD) of four basic ICG waveform feature metrics were studied. These ICG waveform features were measured in the SAICG vector (as the reference errorless value) and in every denoised heartbeat Rq(*k*) vector on a BbyB basis. Thus, a measurement error vector (Err(*k*)) was generated for the feature metric mean absolute error rate% (ER%) accuracy performance using the selected best-performing denoising process as described above. Furthermore, the four ICG feature metrics enable investigating relational characteristics between the feature metrics within the ICG recording mode and between recording modes for the Arm-ICG versus Thorax-ICG [7]. These four basic metrics are labelled A, B, C and VET and defined as follows (refer to Figure 9):

(**A**): For measuring the relative time delay (ms) of the ICG waveform main pulse with respect to the simultaneous ECG R-wave position, namely, (SFP(*k*) − 80), in a particular subject.
(**B**): The ICG waveform main pulse peak amplitude (Ω/s).
(**C**): The ICG main pulse width (ms) at mid-amplitude level (**B**/2).
(**VET**): The ventricular ejection time (ms) used to derive the stroke volume (SV).

In this study, stroke volume (SV) was estimated using Kubicek’s equation [13], which is widely used [5] and has the following expression:

(17) $$\mathrm{SV}=\rho \cdot {\frac{\mathrm{Lo}}{{\mathrm{Zo}}^{2}}}^{2}\cdot {\left(\frac{\mathrm{dZ}}{\mathrm{dt}}\right)}_{\max}\cdot \mathrm{VET}$$

where SV is the estimated stroke volume of every heartbeat (cm^3^); *ρ* is the blood resistivity (typically 150 Ω-cm); Lo is the length of the body tissue impedance [4,7,13], Zo is the ICG Z baseline DC off-set impedance; (dZ/dt)_max_ is the maximum value (Ω/s) of the heartbeat ICG pulse, which occurs after the ECG R-wave; and VET is the ventricular ejection time expressed in seconds (s).

### 2.11. Functional Relationship between Arm-ICG and Thorax-ICG Features Metrics

Following the ICG basic features determination and analysis within a particular ICG recording mode (arm or thorax), the final stage of the study investigated the prospects and extent of considering the Arm-ICG method as a scaled surrogate for the conventional Thorax-ICG, and hence, the potential intrinsic advantages and limitations offered by Arm-ICG methods in ambulatory, long-term cardiac contractility or SV monitoring. Therefore, besides using scatter plots, Pearson correlation (*p*) and linear trend modelling with the associated coefficient of determination (R^2^) [22,23], five functional relationship metrics (**D**, **E**, **F**, **G** and **H**) of Arm- versus Thorax-ICGs, as ratio or difference values, were evaluated and analysed for all subject cases in this study (N = 15) using descriptive statistics. These Arm-Thorax relational metrics were defined as follows:

(**D**): The comparative metric of amplitude B values ratio (%) of Arm(B)/Thorax(B).
(**E**): The time metrics difference (ms) of [Thorax(C) − Arm(C)].
(**F**): The ICG pulse width time metric ratio [Thorax(C)/Arm(C)].
(**G**): The ICG time metrics ratio [Thorax(VET)/Arm(VET)].
(**H**): The ICG volume metric ratio [Thorax(SV)/Arm(SV)].

### 2.12. ICG Signal Frames with Tukey Tapered Cosine Windowing

The BbyB 700 ms ICG frame vectors that had been accurately synchronised with the PQRST events in the ECG were filtered with the various denoising processes under investigation. To minimise the filter transient effects due to abrupt, non-zero signal values at the frame start and end edges, to improve quality of ICG feature extraction, to enable ICG spectral analysis, and to facilitate possible concatenation of the denoised ICG beat frames (joined by zero value segments of respective lengths on a beat-by-beat basis), a 1400-point Tukey tapered cosine windowing vector was multiplied by every 700 ms (1400 sample points) frame vector, as defined in Equation (18) for an M-point Tukey cosine window [39], using Matlab (tukeywin).

(18) $$w\left(x\right)=\left\{\begin{array}{ll} \frac{1}{2}\{1+cos(\frac{2\pi }{2}\left[x-\frac{r}{2}\right])\}, & 0\leq x<\frac{r}{2}\\ 1, & \frac{r}{2}\leq x<1-\frac{r}{2}\\ \frac{1}{2}\{1+cos(\frac{2\pi }{2}\left[x-1+\frac{r}{2}\right])\}, & 1-\frac{r}{2}\leq x\leq 1 \end{array}\right\}$$

where *x* is an *M*-point linearly spaced vector generated using the Matlab linspace command. The parameter *r* is the ratio of the cosine-tapered section length to the entire window length with 0 ≤ *r* ≤ 1; in this study, *r* was set to *r* = 3/14.

### 2.13. Removing Outliers

The resulting data from each of the above-proposed denoising performance metric processing methods and ICG feature metric functional relationships were further processed systematically in order to eliminate outlier values. Statistically, outliers are the data points that differ significantly from other observations, which may cause distortion and skewed data, leading to inaccurate analysis of the results. For the exclusion of such values, output data vectors were further processed in Matlab using the rmoutliers command [22,23,40].

## 3. Results

All ICG recordings were prefiltered with the described bandpass Butterworth and SG filters. Then, three BbyB denoising processes were considered: **(a)** recursive averaging with a 36-beat-sized ensemble averaging buffer (*Nb* = 36), hence the acronym **RAv36**, that was equally applied to both modes of ICG, namely, arm and thorax; **(b)** two-stage Db wavelet filtering of the fourth order for the thorax mode, hence the acronym **WavDb4**, and of the eighth order for the arm mode, hence the acronym **WavDb8**; and **(c)** the cascaded combination of the two denoising processes, depending on the ICG mode, and thus, the acronym ends in 4 or 8: **RAv36 + WavDb4/8**.

### 3.1. ICG Denoising Processes Performance

The ICG denoising performance of the above-indicated advanced filtering processes was assessed using three performance metrics, as mentioned in the Materials and Methods section: (**a**) the SNR (dB) increment, considering Equations (14)–(16), is the difference between the filter output SNR_dB-OUT_ and the input SNR_dB-IN_ on a BbyB basis, and hence, the SNRincre-dB(*k*) denoising performance vector values are in decibels; (**b**) the Pearson correlation coefficient (p) between the denoised ICG incoming beat Rq(*k*) 700 ms frame vector and the SAICG noiseless vector on a BbyB basis, generating the *p*(*k*) denoising performance metric vector; and (**c**) the ICG beat inclusion rate percentage (BIR%) of the heartbeat Rq(*k*) 700 ms vectors with *p* > 0.95, which were counted and divided by the total number of beats (K) in the 480 s ICG recording, then × 100 for the % figure of this denoising performance metric.

Table 5 presents the statistical summary results of the three denoising performance metrics for the three proposed BbyB denoising processes, which were applied to the Thorax-ICG and Arm-ICG recording modes used on the 15 healthy subject cases sample in this study.

**Figure 10:**
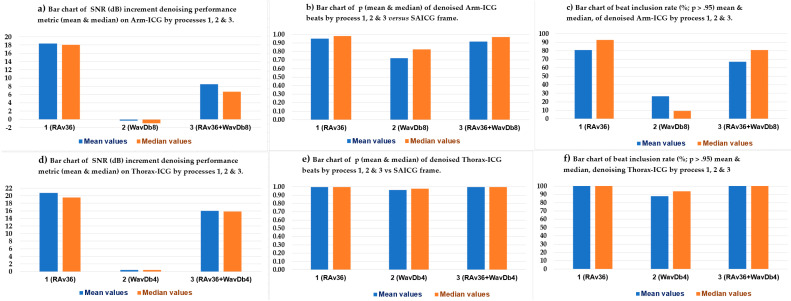
Bar charts for the 3 denoising performance metrics for denoising processes (1), (2) and (3) on the Arm-ICG and Thorax-ICG.

To facilitate the interpretation of the results presented in Table 5, bar charts of the three advanced ICG denoising techniques, namely, (**1**) RAv36, (**2**) WavDb4/8 and (**3**) combined RAv36 + WavDb4/8, performance analysis metrics of the mean and median values in Table 5 are presented in Figure 10a–c for the Arm-ICG recording mode denoising and in Figure 10d–f for the Thorax-ICG recording mode denoising.

From the denoising performance and signal quality enhancement towards the reference signal quality target of the SAICG 700 ms clean signal vector template, of the three BbyB denoising processes in Table 5 and Figure 10, it is evident that the proposed wavelet-based BbyB ICG filtering process displayed relatively poor performance from the three performance metric perspectives. The low performance is also revealed by the *p*(*k*) plot in Figure 8 (black trace) for example case 4. Moreover, the performance analysis results revealed that its cascaded combination with the Rav36 process tended to be detrimental to the denoising process (1). This was so for the more challenging low-amplitude Arm-ICG signals, which had lower initial SNR at the input of the denoising processes, as evidenced in Figure 10a,c. Therefore, the Rav36 process was selected for the following part of this study.

### 3.2. RAv36 Denoised ICG Waveform Features A, B, C and VET Metrics Assessment

#### 3.2.1. Arm-ICG Mode Waveform Features A, B and C Metrics Assessment

The Arm-ICG waveform basic features were the relative delay time position (ms) to the R-wave of the ECG, i.e., metric **A** (see Figure 9); the main Arm-ICG pulse amplitude (Ω/s), metric **B**, which was the maximum of the ICG beat 700 ms frame vector segment from 300 ms to 600 ms; and the Arm-ICG main pulse width at mid-amplitude (**B**/2) level, i.e., metric **C**. Table 6 summarises these Arm-ICG feature metric values and their ER% values.

#### 3.2.2. Thorax-ICG Denoised Waveform Features A, B and C Metrics Assessment

Similarly, for the less challenging stronger ICG signal (about six times larger than in the arm mode), assessment of the denoising capacity of the BbyB ensemble averaging process (RAv36) on the accurate and precise delivery of ICG features metrics A, B and C from the conventional mode Thorax-ICG was implemented using the same feature detection and measurement algorithms as for the arm mode. These results are presented in Table 7. Similarly, the Thorax-ICG signal quality indicators resulting from the beat inclusion filter algorithm, namely, IncBt count, *p*, IncBt and BIR%, are provided for additional consideration.

#### 3.2.3. Arm-ICG vs. Thorax-ICG Waveforms VET Timing Metric Assessment

Estimating the value of VET using non-invasive ICG recording methods is of clinical importance and interest. Therefore, a comparative assessment of the experimental Arm-ICG recording mode vs. the conventional thorax mode was done with regard to clinically relevant parameters, such as the VET feature metric and the resulting estimation of the cardiac stroke volume (SV) metric, as calculated using Kubicek’s Equation (17). Furthermore, the average VET/C ratio within a recording mode was investigated for the sample of 15 healthy volunteers using the C metric results in Table 6 and Table 7. Table 8 summarises the VET metric value descriptive statistics for the arm and thorax modes of the 15 subject cases (N = 15). Moreover, associated ICG signal quality indicators reflected by the BIR% and SNRincre-dB metrics, which resulted from the Rav36 denoising process, are presented there for analysis and results interpretation support information. Furthermore, the case-by-case mean value of the base impedance parameter Zo and the L value for the arm and thorax modes are included in Table 8, which, in combination with the B metric values in Table 6 and Table 7, enable the estimation of the arm and thorax SV metrics for each of the 15 cases and are presented in Table 8.

**Figure 11:**
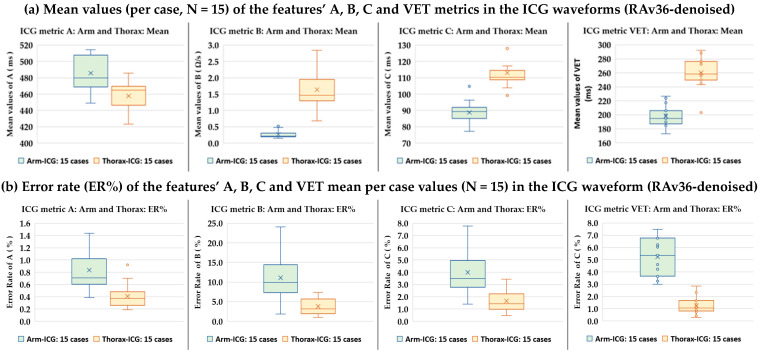
ICG waveform features’ A, B, C and VET measurement values box–whisker chart (created using MS Excel) statistics for the 15 cases presented in Table 6, Table 7 and Table 8: (**a**) mean case values and (**b**) error Rate (ER%) mean value per case for both the Arm-ICG and Thorax-ICG.

To facilitate an appreciation of the level of precision and accuracy of the four main ICG waveform feature metric values results (A, B, C and VET) from Table 6, Table 7 and Table 8, Figure 11 presents, in a familiar way to the medical community, the box-and-whisker chart (Excel MS Office 365) for the metrics’ mean values and respective error rate figures (ER%) per case for the study sample of 15 cases for both the arm (green boxes) and thorax (brown boxes) ICG recording modes. It is remarkable how short the B metric mean box for the Arm-ICG is.

#### 3.2.4. Functional Relationship between Arm-ICG and Thorax-ICG: Metrics D, E, F, G & H

The hypothesised proportionality of the ICG-waveform-based amplitude (Ω/s) and time (ms) feature metrics between the values measured from the conventional Thorax-ICG (control) and from the experimental Arm-ICG as a possible brachial-artery-based surrogate alternative was investigated by means of the five above proposed and defined comparative metrics: D, E, F, G and H (in Section 2.11). These comparative metrics can be derived from the measurement results for metrics B, C, VET, Zo, L and SV extracted from Table 6, Table 7 and Table 8. Table 9 summarises the mean, SD, median and interquartile range (IQR) statistics of the 15 cases for the five comparative metrics between the ICG feature values for the arm versus respective values for the thorax control mode.

**Figure 12:**
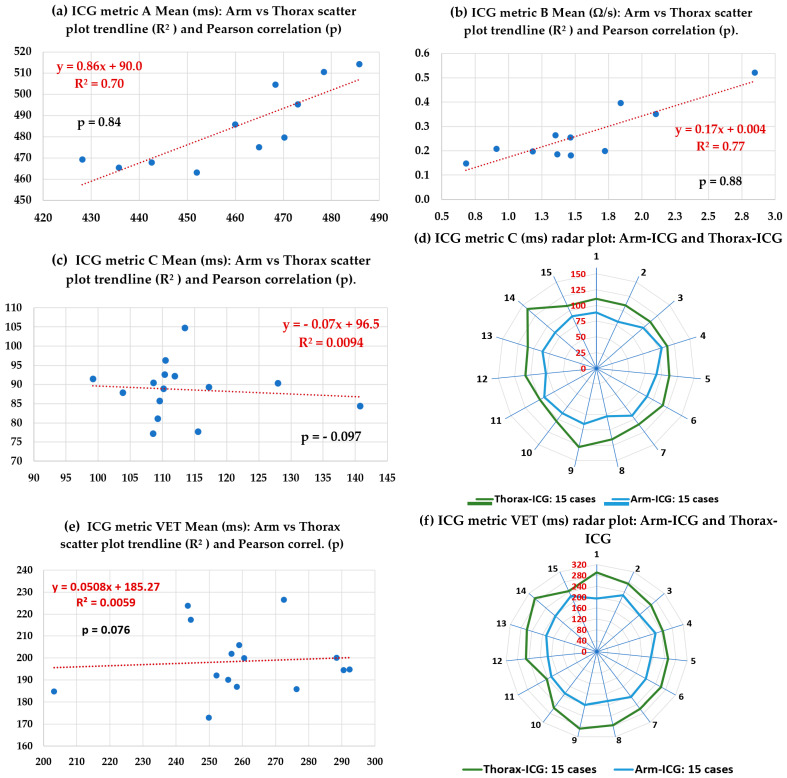
Scatter plots of ICG waveform features’ A, B, C and VET metrics mean values per case (15 subjects) for the Arm-ICG versus Thorax-ICG linear trends analysis, including the R^2^ value and the Pearson correlation (*p*). (**d**,**f**) are radar plots for C and VET.

Furthermore, within this particular investigation task, the functional relationship of the four main feature metrics (A, B, C and VET) between the experimental Arm-ICG method versus the Thorax-ICG control method (standard) from an overall perspective within the 15 healthy subjects sample (N = 15) was analysed by means of the scatter plots presented in Figure 12, including linear trend modelling with the respective coefficient of determination R^2^. Moreover, the calculated Pearson correlation coefficient (*p*) indicator is provided.

The scatter plots of the arm (*y*-axis) versus thorax (*x*-axis) for the A and B metrics shown in Figure 12a,b reveal a reasonable linear (proportionality) functional relationship, with R^2^ > 0.70 and correlation values *p* > 0.80 for N = 15. However, scatter plots (c) and (e) for metrics C and VET, respectively, seem to indicate a clustered relationship trend; the relatively small number of cases (N = 15) presented close timing values of the ICG pulse width (cardiac contraction timing) in healthy subjects, i.e., they were not greatly affected by the subjects’ variations in anatomic or demographic characteristics, mainly body size, weight and age characteristics. Hence, it was important to present these scatter plots to be consistent, objective and provide complementary data in this study. Nevertheless, the respective comparative ratio (proportionality) metrics F and G in Table 9 indicate similar proportionality and were close to unity: F = 1.28 and G = 1.32, with very small SDs. Hence, radial (or radar) plots for metrics C and VET are presented in Figure 12d,f, to support this observed behavior from a different plot type perspective.

### 3.3. Functional Relationship Analysis of Measured SV Values on the Arm-ICG and on the Thorax-ICG

**Figure 13:**
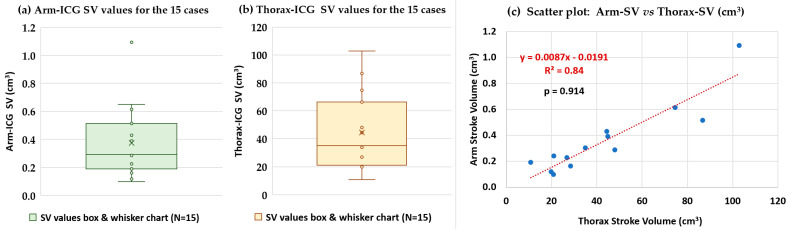
Arm-ICG versus Thorax-ICG functional relationship of estimated stroke volume (SV) values using Kubicek’s formula and ICG waveform metrics after denoising with the RAv36 process and the baseline characteristics of the 15 subject cases.

We undertook further analysis of the hypothesised proportionality hinted at by the comparative metric H in Table 9, i.e., H = 132.8 ± 52.8 (SD), and investigate possible underlying linear trend modelling after an outlier data pair point removal operation [22,23,40]. Figure 13a,b present the box-and-whisker charts of statistical characteristics for the SV values in both ICG recording modes (Arm-SV and Thorax-SV) for the 15 cases, and Figure 13c depicts the scatter plot of the Arm-SV (cm^3^) versus Thorax-SV (cm^3^) in each of the 13 remaining subject case data pair points after the outlier removal operation (two outlier cases), and the Pearson correlation (p) value is indicated. The scatter plot revealed a reliable positive slope linear trend model with R^2^ = 0.84, and the *p* = 0.91 confirmed a good correlation.

## 4. Discussion

This paper presents several key findings. Some were expected and supported the initial hypotheses regarding the performance of methods and the relationship between variables in conventional and experimental ICG recording methods. However, some findings were unexpected, yet still valuable, as they help to mitigate the current knowledge paucity about the effectiveness of arm brachial artery plethysmography (equivalent to an ICG waveform) as a surrogate method for monitoring cardiac contractility [7,8,9]. Using electrodes placed on the upper arm and forearm for ICG recordings to offer a more versatile alternative for long-term ambulatory monitoring has been studied for over a decade [9,16]. However, the success has been limited, largely due to the lack of a robust and effective denoising process, especially on a BbyB basis, to fully exploit the deterministic characteristics of the Arm-ICG signal waveform. This study addressed three main challenges to making Arm-ICG methods a clinically attractive alternative, with the potential to change clinical practice [4,6,7,8,10]. The addressed issues included the following:

(a) Creating an effective real-time prefilter design approach for Arm-ICGs.
(b) Developing a practical, robust, advanced and data-driven BbyB denoising process.
(c) Establishing a compelling functional relationship with the conventional Thorax-ICG waveform feature metrics, which could be used to derive the cardiac SV from Arm-ICG recordings, with satisfactory accuracy and precision.

In this study, a straightforward and highly effective linear IIR Butterworth band-pass filter (ranging between 0.5 and 8 Hz) was found to be useful and necessary, as was also seen in a previous study [7]. Enhancing this initial prefilter stage with a subsequent third-order Savitzky–Golay (SG) linear FIR filter was beneficial. This specific filter was designed according to the procedure described in Section 2.6, making it suitable for the timing characteristics of the ICG signal waveform, particularly the ventricular ejection time (VET) metric. For instance, Figure 10a indicates that the WavDb8 wavelet filter could not denoise the Arm-ICG signal beyond the level achieved by the prefiltering stage (cascaded Butterworth + SG filters). When the WavDb8 denoising process was used on its own, it resulted in a negative mean SNRincre-dB = −0.32 dB, indicating no further improvement in signal quality.

Ensemble averaging in cardiology is a widely accepted and established denoising technique that has been used for the last three decades [24]. It is now incorporated into many commercially available ECG machines and other biomedical signal acquisition devices. Nevertheless, the effectiveness of this process relies heavily on the predictable characteristics of the ECG signal, specifically the timing of the ventricular depolarisation process in the heart. In healthy subjects or most patients presenting sinus rhythm, this ECG feature (reflected in the QRS complex duration feature) is quite deterministic. This was assumed that this was the case with the healthy subjects included in this study, highlighting one of the underlying limitations of ensemble averaging in cardiac signal denoising [24,38,41]. Given this context, an alternative, wavelet-based BbyB denoising process was considered in this study. This was based on previously reported experiences with similar methods in ECG denoising [22,29]. Furthermore, it was hypothesised that the deterministic characteristics of the ventricular depolarisation process were transferred to both the standard Thorax-ICG and the experimental Arm-ICG in this study [7]. This assumption was validated once again here, as demonstrated by the consistent results obtained in this study.

The provision of a reference ICG 700 ms frame vector, or SAICG frame vector, which was assumed to be completely noiseless, as the ICG signal vector from the experimental Arm-ICG recording mode [7] is a powerful research method that helped with answering the questions posed in this study and justified the selective protocol of including only healthy subjects. The noiseless SAICG signal vectors allowed for the performance assessment of the BbyB denoising processes, including the two-stage wavelet-based thresholding optimisation strategy [22], backed by a recursive signal-averaging algorithm. The reference vectors enabled the use of Pearson correlation (*p*) to evaluate the performance of three advanced ICG denoising processes investigated in this study: Rav36, WavDB4/8 and Rav36 + WavDb4/8. Figure 8 clearly shows that the WavDb8 denoising process, which was designed for the Arm-ICG mode, could not stand on its own or in cascaded combination with the recursive averaging RAv36 process in a BbyB ICG denoising approach. Furthermore, the signal reference vectors allowed for defining the noise vector, signal vector and SNRincre-dB denoising performance metric. These definitions confirmed the unsuitability of the WavDb4/8 filter for a proposed BbyB ICG denoising mode, as is evidenced by Table 5 and Figure 10. However, as noted earlier, this finding helps to fill the current knowledge gap about suitable wavelet-based ICG denoising filters applied in a BbyB mode [7,14,17].

Therefore, the refined question of which of the wavelet families would be more suitable for denoising the Arm-ICG signal on a BbyB basis is still open. The fact of the ICG electrodes’ location on the upper arm and the relatively small size of the brachial artery, leading to lower plethysmographic signal power and susceptibility to motion artifact contamination, remains the most challenging issue for obtaining good fidelity Arm-ICG recordings by means of wavelet denoising methods [42] or adaptive filtering techniques, such as least mean squares (LMS) or its normalised approach (NLMS), which were found to be effective for upper arm armband ECG recording [34]. The underlying trade-off issue between the Db and the symlet wavelet families concerns the wavelet symmetry; the Db wavelet has the largest vanishing moment, but at the expense of reducing its symmetry at the same time, whereas the symlet wavelet gains symmetry by reducing the vanishing moment [42]. Nevertheless, it is worth noting that an optimised design of the mother wavelet, which could be effective in retaining T-wave features in a highly noisy ECG denoising process, such as the “fibr” wavelet [42], would be the way forward after taking into consideration the above factors to address the above-refined question for Arm-ICG denoising on a BbyB basis.

The third research task of this study used the “pure” signal waveform vector (700 ms frame) from the SAICG to determine errorless values for the four main ICG waveform feature metrics: A, B, C and VET. The respective four errorless reference metric “true” values allowed for estimating their accuracy performance on their BbyB values by generating corresponding error vectors, namely, Aerr(i), Berr(i), Cerr(i) and VETerr(i), for all the included beats (IncBt), where *p* > 0.95. This accuracy assessment method yielded the respective error rate percentage (ER%) associated with each of the ICG waveform metrics. The results in Table 6, Table 7 and Table 8 show the following average error rate percentages (ER%s) for Arm-ICG and Thorax-ICG: Arm-ICG had 0.83%, 11.1%, 3.99% and 5.2% for A, B, C and VET metrics, respectively; Thorax-ICG had 0.41%, 3.82%, 1.66% and 1.25%, respectively. These ER% values indicate the impact of the RAv36 ICG signal quality enhancement capacity. On **average**, the **overall error rate** achieved with the ICG signals quality enhancement methods in this study was **3.53%** when tested with healthy subjects sitting at rest. Notably, the ER% values were higher for Arm-ICG (mean of 5.28%) compared with Thorax-ICG (mean of 1.79%). The highest ER% was for metric B in the arm due to anatomical variations.

The criteria used in this study for heartbeat ICG inclusion (*p* > *p*_-THRESHOLD_, where the threshold was set to 0.95 in this study) effectively filtered out highly noisy ICG beat events. This method worked as a final filter stage for “gate closing” to noisy ICG beats episodes, or isolated events, that the RAv36 ICG denoising process was unable to sufficiently clean. This approach is especially relevant for real-world, ambulatory long-term Arm-ICG monitoring, where such highly noisy events are expected. The beat inclusion rate% (BIR%) metric is directly related to the chosen *p*_-THRESHOLD_ value and the noise level of the raw ICG input signal. In this study, setting *p*_-THRESHOLD_ > 0.95 resulted in an average BIR% of 80.9% for the Arm-ICG and a clear 100% for the Thorax-ICG. This difference, shown in Table 6 and Table 7, reflects the higher signal quality expected from the conventional Thorax-ICG mode. However, the average BIR% for the arm was not too low, which is encouraging. Furthermore, the BIR% performance metric served as a useful tool for choosing the preferred denoising process, supporting the selection of RAv36, as shown in Figure 10c.

It is worth noting the comparison of mean values and ER% values for the four ICG waveform feature metrics shown in the pairs of box-and-whisker statistics summary charts shown in Figure 11 and Figure 13. Metrics B and VET are particularly crucial for calculating the SV according to Equation (17). Assuming that the parameters L and Zo are accurate, any standard errors in the B and VET values derived from the ICG recording will multiplicatively affect the standard error of the calculated SV. This relationship is evident in Figure 13. Consequently, minimising the ER% for B and VET metrics is crucial to accurately calculate the SV. This is especially true for the Arm-ICG mode, where the B metric has the highest error rate (mean ER% = 11.1%), mostly due to anatomical differences between the subjects, as noted in references [11,12]. Unfortunately, this variation issue cannot be easily corrected. However, it can be appreciated from Figure 11b and Table 8 that the Arm-ICG mode’s VET metric had a relatively large standard deviation (14.2 ms) and ER% (5.2%) values compared with the C metric SD (4.47 ms) and ER% (3.99%), as presented in Table 6. This finding was hypothesised because an algorithm measuring pulse width at the mid-amplitude threshold level (C metric) is likely to be more accurate and precise than an algorithm measuring pulse width at the pulse waveform minimum value (VET metric) in noisy situations. Examining the consistent ratio of VET/C (average value of 2.24), in the Arm-ICG mode (and also in the Thorax-ICG mode) across the 15 cases, as seen in Table 8, suggests a more robust approach. Thus, in the challenging Arm-ICG mode, it might be beneficial to derive the VET metric from the C metric, using the scaling factor of 2.24.

While the sample size in this study was at the pilot study level (a representative small size), limiting the scope for broad clinical generalisation, it is clear that a larger study would be required for that purpose. Despite this limitation, the RAv36 denoising process consistently performed impressively well as a robust and effective BbyB approach throughout the small randomly gathered pilot database of healthy subjects. As evidenced in Table 5 and Figure 10, the proposed method achieved the highest performance metrics for the challenging Arm-ICG mode. These evaluation metrics include a mean SNRincre-dB of 18.4 dB ± 4.6 dB (SD), a mean *p* of 0.952 ± 0.061 (SD) and a mean BIR% of 80.9% ± 24.2% (SD) across the 15 subject cases. This denoising performance level made it possible to establish a promising functional relationship model between the experimental Arm-ICG and the conventional Thorax-ICG, which is demonstrated in Table 9, as well as in the scatter and radar plots in Figure 12. Furthermore, Figure 13 presents a compelling comparison of estimated SV values from the Arm-ICG and Thorax-ICG SV, and their respective scatter plot in Figure 13c reveals a linear trend model. This model can be conveniently expressed in the reverse as

**SV_THORAX_** = (114.94 × **SV_ARM_**) + 2.1954 (19)

with coefficient of determination of **R^2^ = 0.84** and a Pearson correlation of ***p* = 0.914**.

## 5. Conclusions

This research article presents a pilot study for the possible monitoring of ambulatory cardiac contractility from an accessible, comfortable and user-compliant body location by means of wearable sensors technology and bioimpedance plethysmography principles within the vicinity of a major artery vessel; this study also established a functional relationship with the conventional thorax ICG method for SV measurement. The research focused on the possibility of using the left upper arm brachial artery (Arm-ICG) for indirect SV measurement after addressing challenges related to enhancing the quality of the ICG signals on a beat-by-beat basis. This was achieved using a standalone 36-beat (order) recursive ensemble averaging process (RAv36) following a prefiltering process of the raw ICG signal using a linear bandpass of 0.5–8 Hz. An adequate Savitzky–Golay filter design for ICG signals, which was developed using innovative design methods proposed in this study, was used to smooth the data in the prefiltering stage. The ICG denoising process that this study explored can be applied in real time, similar to previous studies undertaken using ECG signals. This study successfully established a functional relationship between ICG waveform features’ metrics from the arm and thorax recording modes. Linear regression trends for Arm-ICG versus Thorax-ICG were analysed and are presented herein, thus addressing the current knowledge gap in this important research line. The determined Arm-ICG time metrics (A, C and VET) had an average error rate of less than 3.3%. The results of this pilot study indicated a clear linear relationship trend between the arm SV and thorax SV, with a high coefficient of determination (R^2^) of 0.84.

## Figures and Tables

**Table 1:** Baseline demographics (15 subjects), of the healthy subjects included in the study: mean, standard deviation (SD), median and interquartile range (IQR) values.

Characteristics	Mean	SD	Median	IQR
Age (y): both genders	38.60	14.45	39.00	23.00
Age (y): females (46.7%)	27.57	6.73	25.00	6.00
Age (y): males (53.3%)	48.25	12.28	48.50	17.75
Height (cm)	167.27	8.52	168.50	10.75
Weight (kg)	70.13	8.75	70.00	14.50
BMI (kg/m^2^)	25.21	3.85	24.65	5.91
Waist measure (cm): around	86.37	12.83	84.00	20.00
Chest measure (cm): around at axilla level (cm)	93.30	7.97	94.00	11.75
Thorax L distance (cm): for estimating SV	39.07	6.91	36.00	12.00
Thorax Zo impedance (Ω): for estimating SV	50.71	9.13	55.50	14.70
Arm circumference (cm): MUAC	8.75	3.85	12.83	7.97
Arm L distance (cm): for estimating SV	16.47	3.66	16.00	3.50
Arm Zo impedance (Ω): for estimating SV	83.30	23.19	91.70	38.85

**Table 2:** Thorax-ICG waveform main heartbeat pulse event timing characterisation by the ventricular ejection time (VET) time in 10 subjects for designing the number of SG samples (N).

Thorax-ICG Cases	1	2	3	4	5	6	7	8	9	10	Mean	SD
**VET** (ms)	257	244	252	261	259	256	203	250	258	243	**248.3**	**16.1**

**Table 3:** Exploratory investigation of the relative process output SNR (dB) performance of Rav16, Rav32 and Rav64 on the standard ECG (Lead I) and Arm-ECG [23].

RecAv(#) Process (# = Beats)	Chest-ECG SNR Mean Value (N = 9)	Chest-ECG SNR Mean Value (dB)	Arm-ECG SNR Mean Value (N = 9)	Arm-ECG SNR Mean Value (dB)	SNRdiff (dB): Thorax-Arm (SNR_dB_)
RecAv(16)	106.3	40.5	13.1	22.3	18.2
RecAv(32)	194.4	45.8	39.4	31.9	13.9
RecAv(64)	256.0	48.2	64.4	36.2	12.0

**Table 4:** Wavelet optimal sigma (σ_optim_) thresholding values (in µV) for the Db4 (Thorax-ICG) and Db8 (Arm-ICG) wavelet denoising process, computed for each subject case in the study.

Case # →	1	2	3	4	5	6	7	8	9	10	11	12	13	14	15
Thorax-ICG Db4 σ_optim_ (µV)	16	20	10	7.0	55	14	18	7.0	5.0	22	28	10	9.0	24	12
Arm-ICG Db8 σ_optim_ (µV)	36	20	60	18	16	85	24	60	9.0	25	60	55	45	54	6.5

**Table 5:** ICG denoising processes performance on the Thorax-ICG and Arm-ICG, showing the mean (±SD) and median (IQR) of the SNR (dB) increment of denoised ICG beats from the raw input ICG SNR, taking the SAICG “noiseless” vector reference as the ICG signal vector (**SNRincre-dB**); Pearson correlation (*p*) of the denoised ICG beats versus the SAICG noiseless vector; and ICG beat inclusion rate (**BIR%**) with *p* > 0.95 out of the total beats (K) for the sample size of N = 15 in this study.

Performance Metric on ICG	RAv36	WavDb4/8	RAv36 + WavDb4/8
SNRincre-dB mean (±SD): Thorax-ICG, all beats	20.72 (±4.69)	0.42 (±0.89)	16.03 (±4.38)
SNRincre-dB mean (±SD): Arm-ICG, all beats	18.36 (±4.59)	−0.32 (±1.47)	8.54 (±6.91)
SNRincre-dB median (IQR): Thorax-ICG, all beats	19.56 (4.61)	0.42 (0.71)	15.84 (5.81)
SNRincre-dB median (IQR): Arm-ICG, all beats	18.06 (6.61)	−0.94 (1.44)	6.71 (8.08)
*p* mean (±SD): Thorax-ICG, all beats	0.998 (±0.0018)	0.963 (±0.0330)	0.998 (±0.0022)
*p* mean (±SD): Arm-ICG, all beats	0.952 (±0.0607)	0.721 (±0.206)	0.919 (±0.1050)
*p* median (IQR): Thorax-ICG all beats	0.999 (0.0016)	0.978 (0.0359)	0.998 (0.0019)
*p* median (IQR): Arm-ICG all beats	0.982 (0.0387)	0.824 (0.318)	0.9701 (0.0845)
BIR% mean (±SD): Thorax-ICG (BbyB)	100.0 (±0.0)	88.01 (±13.06)	100.0 (±0.00)
BIR% mean (±SD): Arm-ICG (BbyB)	80.89 (±24.17)	26.69 (±29.95)	67.31 (±32.95)
BIR% median (IQR): Thorax-ICG (BbyB)	100.0 (0.0)	93.62 (10.92)	100.0 (0.00)
BIR% median (IQR): Arm-ICG (BbyB)	92.86 (25.59)	9.46 (42.13)	81.12 (47.66)

**Table 6:** Arm-ICG RAv36 denoised waveform metrics A, B and C: mean, SD, median, IQR and mean absolute error rate (ER%) for the included ICG beats (IncBt) presenting a Pearson coefficient *p* > 0.95 for BbyB versus the SAICG frame, as well as the associated BIR% metric.

Case #	IncBt Count (*p* > 0.95)	p IncBt Mean	IncBt BIR% (%)	A Mean (ms)	A SD (ms)	A Medn (ms)	A IQR (ms)	A ER% (%)	B Mean (Ω/s)	B SD (Ω/s)	B Medn (Ω/s)	B IQR (Ω/s)	B ER% (%)	C Mean (ms)	C SD (ms)	C Medn (ms)	C IQR (ms)	C ER% (%)
**1**	462	0.991	93.5	466	3.21	466	3.50	0.520	0.483	0.052	0.472	0.068	8.49	88.9	3.43	89.0	5.00	3.03
**2**	120	0.968	20.9	469	5.48	470	8.50	1.082	0.214	0.046	0.210	0.087	19.0	81.2	8.31	78.5	9.13	7.77
**3**	393	0.984	64.5	511	7.93	509	10.5	1.351	0.151	0.026	0.150	0.024	14.2	96.3	6.00	96.0	7.00	4.71
**4**	639	0.992	100	514	5.79	517	9.50	0.957	0.199	0.024	0.200	0.032	9.90	104.8	5.78	104.0	10.0	4.55
**5**	386	0.988	92.1	514	4.13	515	4.00	0.597	0.521	0.069	0.511	0.120	10.8	92.3	4.61	91.5	4.50	3.48
**6**	272	0.968	50.9	475	6.54	475	11.0	1.437	0.208	0.067	0.182	0.109	24.1	89.3	7.08	89.8	10.5	6.20
**7**	394	0.986	91.8	486	4.31	487	6.00	0.709	0.264	0.023	0.266	0.029	6.64	92.6	3.25	93.0	3.50	2.73
**8**	188	0.972	46.5	449	6.37	449	10.5	1.206	0.181	0.037	0.185	0.051	18.1	77.8	4.68	76.8	5.50	4.60
**9**	542	0.993	98.7	468	4.12	469	5.00	0.682	0.199	0.021	0.192	0.035	8.88	90.4	3.01	90.0	4.50	2.83
**10**	455	0.984	93.1	513	5.33	513	10.0	0.948	0.352	0.024	0.346	0.034	5.40	88.0	2.52	87.5	2.50	2.28
**11**	400	0.980	76.3	479	4.74	479	7.00	0.809	0.186	0.024	0.183	0.032	10.3	91.6	6.67	90.0	9.00	5.91
**12**	444	0.986	93.7	495	3.48	496	5.50	0.608	0.265	0.019	0.267	0.023	6.17	77.2	2.63	77.5	4.00	2.94
**13**	624	0.994	100	505	3.47	505	4.50	0.536	0.255	0.025	0.254	0.034	8.02	85.8	2.45	85.5	3.50	2.23
**14**	486	0.984	98.4	480	4.54	480	5.00	0.695	0.149	0.028	0.146	0.035	14.7	84.5	5.13	84.8	8.50	5.22
**15**	468	0.998	92.9	463	2.26	463	3.00	0.388	0.397	0.008	0.397	0.010	1.83	90.5	1.48	90.5	2.00	1.40
Mean	-->	0.98	80.9	485.8	4.78	486	6.90	0.83	0.27	0.03	0.26	0.05	11.1	88.7	4.47	88.3	5.94	3.99

**Table 7:** Thorax-ICG RAv36 denoised waveform metrics A, B and C: mean, SD, median, IQR and mean absolute error rate (ER%) for the included ICG beats (IncBts), presenting a Pearson coefficient *p* > 0.95 for BbyB versus the SAICG frame.

Case #	IncBt Count (*p* > 0.95)	*p* IncBt Mean	IncBt BIR% (%)	A Mean (ms)	A SD (ms)	A Medn (ms)	A IQR (ms)	A ER% (%)	B Mean (Ω/s)	B SD (Ω/s)	B Medn (Ω/s)	B IQR (Ω/s)	B ER% (%)	C Mean (ms)	C SD (ms)	C Medn (ms)	C IQR (ms)	C ER% (%)
**1**	494	0.999	100	436	1.32	436	1.50	0.25	1.24	0.027	1.24	0.034	1.66	110	1.98	110	2.50	1.42
**2**	619	0.997	100	428	2.82	429	3.00	0.25	2.05	0.182	2.08	0.138	5.66	109	3.61	110	3.00	2.17
**3**	612	0.997	100	478	3.25	479	3.50	0.49	1.86	0.083	1.85	0.138	3.82	110	1.73	111	1.50	1.12
**4**	634	0.999	100	486	1.86	486	2.00	0.31	1.72	0.022	1.72	0.030	1.04	113	1.36	114	1.50	0.89
**5**	434	0.997	100	469	2.84	469	3.50	0.48	2.85	0.229	2.91	0.322	6.93	112	4.01	111	3.50	2.34
**6**	544	0.995	100	465	4.18	466	5.00	0.70	0.91	0.107	0.91	0.088	7.39	117	5.23	117	5.63	3.30
**7**	319	0.999	100	460	2.09	460	3.00	0.37	1.35	0.054	1.36	0.072	3.14	110	1.49	110	2.50	1.08
**8**	419	10.00	100	451	1.13	451	1.00	0.19	1.47	0.038	1.45	0.065	2.24	115	0.67	116	1.00	0.46
**9**	539	0.999	100	443	2.85	443	3.50	0.49	1.18	0.044	1.18	0.077	3.18	128	3.60	127	5.00	2.29
**10**	444	0.998	100	468	2.18	468	2.50	0.41	2.10	0.028	2.10	0.039	1.10	104	2.30	104	3.00	1.74
**11**	554	0.999	100	423	1.39	424	1.50	0.24	1.36	0.091	1.33	0.107	5.50	99	1.81	99	2.38	1.40
**12**	484	0.998	100	473	2.33	473	3.00	0.39	2.50	0.055	2.49	0.044	1.57	109	0.97	109	1.00	0.70
**13**	564	0.999	100	468	1.52	469	2.50	0.27	1.46	0.100	1.42	0.138	5.67	110	2.32	109	3.50	1.75
**14**	504	0.993	100	470	5.40	470	7.00	0.92	0.68	0.052	0.68	0.061	5.90	141	5.84	141	8.50	3.43
**15**	452	0.999	100	452	1.83	452	3.00	0.34	1.84	0.053	1.85	0.080	2.51	109	1.12	109	1.50	0.78
Mean	-->	0.998	100	458	2.47	458	3.03	0.41	1.64	0.08	1.64	0.10	3.82	113	2.54	113	3.07	1.66

**Table 8:** Ventricular ejection fraction time (VET) metric values and comparative VET/C ratio, Zo, L value and calculated SV per subject (N = 15) in the arm and thorax RAv36-denoised ICGs.

	Case # →	1	2	3	4	5	6	7	8	9	10	11	12	13	14	15	Mean
**Arm-ICG**	**BIR%** at *p* > 0.95 (%)	93.5	20.9	64.5	100	92.1	50.9	91.8	46.5	98.7	93.0	76.3	93.7	100	98.4	92.9	**80.9**
	**SNRincre-dB** mean (dB)	19.0	23.3	26.1	18.1	16.7	13.4	15.8	17.4	12.6	10.4	22.1	19.8	24.5	21.7	14.7	**18.4**
	**VET** mean (ms)	194	227	202	217	192	200	206	186	200	190	185	173	187	195	224	**198**
	**VET** SD (ms)	18.5	22.3	9.73	7.79	10.4	15.5	17.7	16.1	11.3	10.7	17.9	22.2	12.6	13.2	7.30	**14.2**
	**VET** median (ms)	191	229	202	218	192	201	207	182	198	190	182	168	186	194	225	**198**
	**VET** IQR (ms)	14.0	17.3	11.5	12.5	14.4	17.6	19.3	16.5	17.5	8.50	14.5	12.8	9.50	16.8	11.0	**14.2**
	**VET** ER% (%)	6.03	6.87	3.65	3.00	4.36	6.78	7.49	6.22	4.60	4.22	6.15	6.76	3.63	5.35	3.25	**5.2**
	**VET/C** (ratio)	2.19	2.79	2.10	2.07	2.08	2.24	2.22	2.39	2.21	2.16	2.02	2.24	2.18	2.31	2.47	**2.24**
	**Base imp. Z_o_** mean (Ω)	76.4	60.2	93.6	46.7	116	91.7	54.9	54.5	57.9	113	109	97.1	92.3	98.7	87.3	**83.3**
	**L_ARM_** (cm)	10.0	18.0	15.0	12.0	16.0	16.0	12.0	19.0	17.0	17.0	15.0	20.0	19.0	19.0	25.0	**16.7**
	**SV** (cm^3^)	0.24	0.65	0.12	0.43	0.29	0.19	0.39	0.61	0.51	0.23	0.10	0.29	0.30	0.16	1.09	**0.37**
**Thorax-ICG**	**BIR%** at *p* > 0.95 (%)	100	100	100	100	100	100	100	100	100	100	100	100	100	100	100	**100**
	**SNRincre-dB** mean (dB)	21.1	19.6	12.7	28.5	16.0	18.3	21.5	23.9	17.0	28.9	19.6	27.6	17.9	19.4	18.9	**20.7**
	**VET** mean (ms)	291	273	257	244	252	261	259	276	288	256	203	250	258	292	243	**260**
	**VET** SD (ms)	1.22	8.53	6.45	3.39	3.48	10.92	3.03	2.24	4.42	1.30	2.19	2.78	5.29	12.97	2.85	**4.74**
	**VET** median (ms)	291	274	259	244	252	262	259	277	288	256	204	250	259	297	244	**261**
	**VET** IQR (ms)	1.50	2.50	4.50	4.00	4.50	10.0	5.00	3.50	7.50	1.50	3.50	3.00	6.00	10.5	3.50	**4.73**
	**VET** ER% (%)	0.31	1.68	1.73	1.06	1.11	2.84	1.01	0.72	1.35	0.42	0.88	0.83	1.65	2.31	0.91	**1.25**
	**VET/C** (ratio)	2.64	2.49	2.32	2.15	2.25	2.22	2.35	2.39	2.25	2.46	2.05	2.30	2.36	2.08	2.24	**2.30**
	**Base imp. Z_o_** mean (Ω)	56.0	56.6	60.7	42.9	56.9	57.8	38.9	38.8	37.6	55.5	66.2	46.4	56.0	49.1	41.2	**50.7**
	**L_THORX_** (cm)	35.0	36.0	32.0	36.0	38.0	32.0	36.0	31.0	49.0	32.0	47.0	39.0	44.0	48.0	51.0	**39.1**
	**SV** (cm^3^)	21.1	33.8	19.9	44.4	48.0	10.9	44.9	74.7	86.7	26.8	21.0	66.2	35.0	28.5	103	**44.3**

**Table 9:** Summary of the overall (N = 15) Arm-ICG and Thorax-ICG comparative metrics D, E, F, G and H, which were derived based on their definitions in Section 2.11 and the results in Table 6, Table 7 and Table 8.

Comparative Metric	Mean	SD	Median	IQR
**D = [Arm(B)/Thorax(B)] (%)**	17.4	7.51	16.8	8.63
**E** **= [Thorax(C) − Arm(C)] (ms)**	24.4	12.7	21.3	12.9
**F** **= [Thorax( C)/Arm(C)] (ratio)**	1.28	0.16	1.24	0.19
**G** **= [Thorax(VET)/Arm(VET)] (ratio)**	1.32	0.14	1.31	0.21
**H = [Thorax(SV)/Arm(SV)] (ratio)**	132.8	52.8	118.7	70.6

## Data Availability

Research data related to this article is available in the Ulster University PURE repository portal under the name of the corresponding author’s research output in the datasets area: https://pure.ulster.ac.uk/en/persons/omar-escalona/publications/ (accessed on 15 May 2023).
